# Protective effects of dog ownership against the onset of disabling dementia in older community-dwelling Japanese: A longitudinal study

**DOI:** 10.1016/j.pmedr.2023.102465

**Published:** 2023-10-07

**Authors:** Yu Taniguchi, Satoshi Seino, Tomoko Ikeuchi, Toshiki Hata, Shoji Shinkai, Akihiko Kitamura, Yoshinori Fujiwara

**Affiliations:** a Japan Environment and Children’s Study Programme Office, National Institute for Environmental Studies, Tsukuba, Japan. ADRESS:16-2 Onogawa, Tsukuba, Ibaraki 305-8506, Japan; b Research Team for Social Participation and Community Health, Tokyo Metropolitan Institute of Gerontology, Tokyo, Japan. ADRESS: 35-2 Sakae-cho, Itabashi-ku, Tokyo 173-0015, Japan; c Human Care Research Team, Tokyo Metropolitan Institute of Geriatrics and Gerontology, Tokyo, Japan. ADRESS: 35-2 Sakae-cho, Itabashi-ku, Tokyo 173-0015, Japan; d Faculty of Nutrition, Kagawa Nutrition University, Saitama, Japan. ADRESS: 3-9-21 Chiyoda, Sakado City, Saitama 350-0288, Japan

**Keywords:** Animals, Dog, Dementia, Exercise, Aging

## Abstract

This prospective study examined the associations of dog/cat ownership with incident disabling dementia using propensity score matching based on the physical, social, and psychological characteristics of dog and cat owners. We also examined associations of the interaction between dog/cat ownership and exercise habit and social isolation with dementia. Overall, 11,194 older adults selected using stratified and random sampling strategies in 2016 were analyzed. Dog/cat ownership was defined as “current” or “past and never”. Disabling dementia was defined according to physicians’ rating in the long-term care insurance system in Japan during the approximately 4-year follow-up period. Statistical analysis was weighted by the inverse of the propensity score in the generalized estimating equation after adjusting for follow-up period. Current dog owners (8.6 %) had an odds ratio (OR) of 0.60 (95 %CI: 0.37–0.977) of having disabling dementia compared to past and never owners. For cat ownership, the corresponding OR was 0.98 (95 %CI: 0.62–1.55). Current dog owners with a regular exercise habit had an OR of 0.37 (0.20–0.68) compared to past and never dog owners with no exercise habit. Further, current dog owners with no social isolation had an OR of 0.41 (0.23–0.73) compared to past and never dog owners with social isolation. Dog ownership had a suppressive effect on incident disabling dementia after adjusting for background factors over a 4-year follow-up period. Specifically, dog owners with an exercise habit and no social isolation had a significantly lower risk of disabling dementia.

## Highlights

• Dog ownership has a suppressive effect on incident disabling dementia.
• Dog owners with exercise habit and no social isolation have significantly lower risk.
• Whereas cat ownership was not effective for preventing dementia.

## 1 Introduction

Epidemiological literature on pet ownership has reported several potential health benefits, including improved activities of daily living (Raina et al., 1999), mortality (Adhikari et al., 2019, Mubanga et al., 2017), and cumulative survival rates (Hoshi et al., 2017). Although some previous study reported no-significant association of pet ownership with mortality (Christopoulos et al., 2022, Gillum and Obisesan, 2010), systematic review and meta-analysis concluded that dog ownership is associated with lower risk of death over the long term (Kramer et al., 2019). Our previous study revealed that older adults who had owned a dog and/or cat had lower risk of incident frailty, with dog owners at baseline showing 0.81 times the likelihood of developing incident frailty than never dog owners during 2-year follow-up period (Kojima et al., 2020, Taniguchi et al., 2019). We also reported that dog owners at baseline had an odds ratio (OR) of 0.54 of disability onset during an approximately 3.5-year follow-up period (Taniguchi et al., 2022). These findings suggest that dog ownership among older adults has a protective effect on frailty, and disability and all-cause mortality.

Dementia is known as a prognostic factor of frailty (Kojima et al., 2016), and is the main cause of disability (Lisko et al., 2021)and a leading cause of mortality (Collaborators, 2021) in old age. In a recent study, we proposed that regular exercise interacts with dog ownership to reduce the risk of disability (Taniguchi et al., 2022). Physical activity, including having an exercise habit, is protectively linked to reduced incident cognitive decline and dementia (Blondell et al., 2014, Ihira et al., 2022, Larson et al., 2006, Laurin et al., 2001). In addition to physical inactivity, Livingston et al. reported that social isolation is also a potential modifiable risk factor for dementia (Livingston et al., 2017). Therefore, we hypothesized that dog ownership may have a protective effect against incident dementia through the presence of a regular exercise habit and absence of social isolation, which could explain the mechanism for frailty, disability, and all-cause mortality. However, available evidence is insufficient to confirm this hypothesis.

Despite the difficulty of examining the direct health effects of dog and cat ownership, research methods based on propensity score matching have recently been used in epidemiological studies (Iwagami and Shinozaki, 2022). In this study, we used propensity score matching based on available evidence, namely, the physical, social, and psychological characteristics of community-dwelling elderly Japanese dog and cat owners (Taniguchi et al., 2018b). This study had two objectives: to identify the effects of dog/cat ownership on the risk of incident disabling dementia, net of baseline health, and to examine the associations of the interaction between experience with dog/cat ownership and exercise habit and social isolation with incident disabling dementia. This study yields new insights into strategies to promote health in older adults.

## 2 Methods

### 2.1 Study population

Data for the study were collected as part of a community-wide intervention trial (the Ota Genki Senior Project) launched in 2016. Details of the study design are reported elsewhere (Seino et al., 2018a, Seino et al., 2018b, Seino et al., 2019, Seino et al., 2021). Briefly, 15,500 residents aged 65–84 years comprising approximately 10 % of the older population of Ota City, Tokyo, Japan, were selected using stratified and random sampling strategies in all 18 districts. In June 2016 we mailed a self-administered questionnaire that constituted the baseline survey to the 15,500 residents; 11,925 questionnaires were returned (response rate 76.9 %). All participants were physically and cognitively independent, defined as the absence of long-term care insurance certification (Ministry of Health, 2002). Among the 11,925 subjects, 11,233 (rate of valid responses, 72.5 %) completed the questionnaire on experience with dog/cat ownership (Taniguchi et al., 2018b). We checked for incident disabling dementia until July 2020 using data from the long-term care insurance system (LTCI) (Taniguchi et al., 2017a, Taniguchi et al., 2018a); thus, the follow-up period was approximately 4 years.

### 2.2 Eligibility criteria

To be eligible for the study, individuals had to have received an assessment for incident disabling dementia. A total of 39 participants were excluded because of logical contradiction of data. Thus, we received complete data from 11,194 participants (rate of valid responses, 72.2 %). All data collection in the Ota Genki Senior Project was performed in accordance with the relevant guidelines of the Ethical Committee of the Tokyo Metropolitan Institute of Geriatrics and Gerontology. We adhered strictly to the Declaration of Helsinki. This study was approved by the Ethics Committee of the Tokyo Metropolitan Institute of Geriatrics and Gerontology. A statement attached to the questionnaire explained the purpose of the survey, the voluntary nature of participation, and promised anonymity in the analysis. Returning the questionnaire was taken to indicate consent to participate.

### 2.3 Definition of dog/cat ownership

Participants were asked if they lived with a pet (current, past, or never). Those with current or past pet ownership experience were asked to indicate the pet species (dog, cat, or other) (Taniguchi et al., 2022, Taniguchi et al., 2018b, Taniguchi et al., 2019). These responses were used to classify dog ownership and cat ownership as “current” or “past and never” in the baseline survey.

### 2.4 Measurement of dementia

The Japanese LTCI system was established to support the need for long-term care services, community-based services, and in-facility services. All primary insured persons aged 65 years or older are candidates for care, and secondary insured persons aged 40–64 years with any of 15 specific diseases can also utilize care services. The long-term care approval board investigates the mental and physical condition of the applicants and make a screening judgement based on the opinions of physician. As part of the LTCI system, which covers most individuals with dementia, the Ministry of Health, Labor and Welfare of Japan requires that physicians provide an observer-based rating for older adults with dementia (Nakanishi and Nakashima, 2014). The physician’s rating is a standard form used to assess patients’ chronic medical conditions and functions of daily life (Tomata et al., 2016), and is used throughout the country (Nakazawa et al., 2012). The outcome categories of the scale are: no dementia; some dementia, but almost independent in daily life (level I); dementia with some difficulty communicating, but independent in daily living with minimal observation (level II); dementia with some difficulty communicating and a need for partial care (level III); and severe dementia with difficulty communicating and a need for complete care (level IV). In the present study, disabling dementia was defined as a classification of level II or higher (Taniguchi et al., 2017a, Taniguchi et al., 2017b, Tomata et al., 2016, Taniguchi et al., 2019), as this is the level at which applicants are entitled to receive insurance benefits, including institutional, home, respite and/or day care, and loans of equipment (Ikeda et al., 2008, Ministry of Health, Labour and Welfare, 2016, Yamamoto et al., 2012). We extracted the date on which participants received a disabling dementia classification in the LTCI system.

### 2.5 Other variables

Covariates included the following sociodemographic characteristics: sex, age, household size, marital status, educational attainment, equivalent income, employment, history of chronic diseases, history of hospitalization during the past year, fall during the past year, alcohol consumption, smoking status, sleep duration, food variety, Tokyo Metropolitan Institute of Gerontology Index of Competence [TMIG-IC] score, mobility limitation, body mass index, Motor Fitness Scale, exercise habit, frailty status, interaction with neighbors, social isolation, trust in neighbors, frequency of going outdoors, subjective happiness, self-rated health, Geriatric Depression Scale [GDS]-5 score, and World Health Organization Five [WHO-5] Well-Being Index. Chronic diseases included the following clinically relevant medical conditions: hypertension, hyperlipidemia, heart disease, stroke, diabetes mellitus, bone and joint disease, lung respiratory disease, and cancer. For each of these conditions, participants were asked if they had received a physician diagnosis (yes or no). Food variety was assessed using the dietary variety score, calculated based consumption frequencies for 10 food items (meat, fish/shellfish, eggs, milk, soybean products, green/yellow vegetables, potatoes, fruit, seaweed, and fats/oils) during the week (Kumagai et al., 2003). The score ranges from 0 to 10, with higher scores indicating greater food variety. The TMIG-IC was designed to measure higher-level functioning in older community residents (Koyano et al., 1991). The score ranges from 0 to 13, with lower scores indicating lower functional capacity. Mobility limitation was defined as self-reported difficulty walking one-quarter of a mile or climbing 10 steps without resting. The Motor Fitness Scale was evaluated based on 14 items on basic motor ability. The score ranged from 0 to 14, with higher scores indicating greater motor ability. Since higher physical activity is one of the characteristics of pet owners (Coleman et al., 2008), we assessed exercise habit. Participants were asked about the types of exercise they engaged in more than once per week: no exercise, walking, running, muscle training, stretching, swimming, cycling, yoga exercises, and other. Participants were then classified as having a regular exercise habit or no regular exercise habit (Taniguchi et al., 2022). Frailty status was assessed using a modified version of the Kaigo-Yobo Checklist (Shinkai et al., 2010). The score ranges from 0 to 15, and a score higher than 4 was defined as frailty. Interaction with neighbors was classified as a close relationship, conversation level, exchange of greetings only, or no social contact. Social isolation was assessed based on the frequencies of face-to-face and non-face-to-face contact with non-resident children, relatives and friends, or neighbors. An overall frequency of contact with others of less than once a week was categorized as social isolation. The WHO-5 is designed to measure psychological health. The WHO-5 score ranges from 0 to 100, with lower scores indicating lower positive mood and vitality.

### 2.6 Statistical analysis

First, we determined participants’ baseline demographic and health characteristics according to experience with dog and cat ownership. Second, to examine the effects of dog and cat ownership on the risk of incident disabling dementia, net of baseline health, an inverse probability of treatment weighted logistic regression model with the propensity score was implemented. This model enabled us to balance baseline characteristics in current owners and past and never owners by weighting each individual in the analysis. Standard error of the estimated value for experience with dog and cat ownership and incident disabling dementia was calculated with robust variance. Weight was calculated based on the physical, social, and psychological characteristics of community-dwelling elderly Japanese dog and cat owners (Taniguchi et al., 2018b). For dog ownership, weight was calculated based on sex, age, household size, marital status, educational attainment, equivalent income, employment, history of chronic diseases (lung respiratory disease, and cancer), history of hospitalization during the past year, fall during the past year, alcohol consumption, smoking status, TMIG-IC score, mobility limitation, motor fitness scale, frailty status, interaction with neighbors, social isolation, trust in neighbors, subjective happiness, and WHO-5. For cat ownership, weight was calculated based on sex, age, household size, marital status, educational attainment, equivalent income, employment, fall during the past year, smoking status, TMIG-IC score, interaction with neighbors, and social isolation. Follow-up period was adjusted. We also examined associations of the interaction for experience with dog/cat ownership and regular exercise habit and social isolation with incident disabling dementia by an inverse probability of treatment weighted logistic regression model with the propensity score, adjusted for follow-up period. Odds ratio > 1 indicates that the current owners had a lower risk with incident disabling dementia than past and never owners. Statistical analyses were conducted using SPSS (version 23.0; IBM Corp, Armonk, NY, USA).

## 3 Results

### 3.1 Sample characteristics

Data from the baseline survey of 11,194 participants showed that the mean (SD) age of participants was 74.2 (5.4) years, and 51.5 % were women. The average household size was 2.3 (1.1), 67.1 % were married, 63.2 % had an educational attainment of below high school level, and 66.0 % had an equivalent income of less than 4,000,000 yen (USD26,869 on Oct 2022). 959 participants (8.6 %) and 704 participants (6.3 %) were current dog and cat owners and 10,235 participants (91.4 %) and 10,490 participants (93.7 %) were past or never (non-current) dog and cat owners, respectively. Among current owners, 124 participants owned both of dog and cat. Table 1 shows the baseline demographic and health characteristics of dog and cat owners.

**Table 1:** Baseline demographic and health characteristics in older community-dwelling Japanese by dog and cat ownership experience.

Variable	Experience with dog ownership		Experience with cat ownership	
	Current (n = 959, 8.6 %)	Past and never (n = 10235, 91.4 %)	Current (n = 704, 6.3 %)	Past and never (n = 10490, 93.7 %)
Sex (%, female)	54.4	51.2	52.1	51.5

Age (%)				
65–74 years	59.2	46.7	60.4	47.0
75–84 years	40.8	53.3	39.6	53.0

Household size	2.7 (1.2)	2.2 (1.1)	2.6 (1.1)	2.3 (1.1)

Marital status (%)				
Married	74.0	66.5	73.9	66.7
Divorced	5.8	6.1	5.7	6.1
Widowed	18.1	19.7	16.3	19.8
Single	2.1	7.7	4.2	7.4
Educational attainment (%)				
Elementary school	0.8	1.5	1.2	1.5
Middle school	17.2	23.8	19.9	23.5
High school	37.6	38.6	35.4	38.7
College, university, or graduate school	42.1	34.2	41.6	34.4
Other	2.2	1.9	2.0	1.9

Equivalent income (%)				
<1,000,000yen	6.5	7.3	5.4	7.4
1,000,000yen-2,500,000yen	26.2	33.6	28.8	33.3
2,500,000yen-4,000,000yen	22.7	26.0	26.7	25.7
≥4,000,000yen	26.4	19.0	24.0	19.3
Unknown	18.1	14.0	15.1	14.3

Employment (%, present)	35.6	27.3	36.0	27.4

Chronic disease (%)				
Hypertension	55.2	54.3	51.2	54.5
Hyperlipidemia	44.6	42.1	44.0	42.2
Heart disease	21.3	22.0	20.4	22.1
Stroke	8.1	7.6	9.1	7.5
Diabetes mellitus	18.3	18.7	18.0	18.7
Bone and joint disease	29.8	31.9	30.7	31.8
Lung respiratory disease	13.5	15.3	14.9	15.1
Cancer	17.8	16.6	17.7	16.6

Hospitalization during the past year (%)	14.7	12.5	12.5	12.7
Fall during the past year (%)	15.3	15.0	14.7	15.0
Alcohol consumption status (%)				
Current	57.4	54.8	55.9	55.0
Past	5.9	8.4	8.4	8.1
Never	36.7	36.8	35.7	36.8

Smoking status (%)				
Current	14.0	12.4	16.2	12.3
Past	33.8	32.7	33.3	32.8
Never	52.2	54.8	50.4	54.9

Sleep duration (min)	396 (69)	396 (74)	397 (74)	396 (73)
Food variety (score)	3.0 (2.2)	3.2 (2.2)	3.2 (2.2)	3.2 (2.2)
TMIG-IC (score)	11.6 (1.8)	11.4 (1.9)	11.4 (2.0)	11.4 (1.9)
Mobility limitation (%)	25.3	29.7	28.3	29.4
BMI (kg/m^2^)	23.0 (3.3)	22.7 (3.2)	22.7 (3.3)	22.7 (3.2)
Motor fitness scale (score)	11.0 (3.1)	10.6 (3.3)	10.8 (3.2)	10.6 (3.3)
Regular exercise habit (%)	77.8	73.5	70.1	74.1
Frailty (%)	20.8	23.9	23.2	23.7

Interaction with neighbors (%)				
Significant relationship	25.5	23.1	23.6	23.2
Conversation	40.6	37.1	40.4	37.2
Exchange of greetings only	29.7	32.9	31.0	32.7
No social contact	4.2	6.9	5.0	6.8
Social isolation (%)	23.9	32.7	29.6	32.1
Trust in neighbors (%, yes)	81.1	76.8	81.2	76.9

Frequency of going outdoors (%)				
At least once a day	81.2	74.2	76.8	74.6
Once every 2–3 days	14.6	18.5	14.0	18.5
Less than once a week	4.2	7.3	9.2	6.9

Subjective happiness (%)				
Happy, rather happy	95.0	93.6	96.1	93.5
Rather unhappy, unhappy	5.0	6.4	3.9	6.5

Self-rated health (%)				
Excellent, good	83.2	81.0	83.7	80.9
Fair, poor	16.8	19.0	16.3	19.1

GDS Short-version (score)	1.2 (1.2)	1.3 (1.3)	1.3 (1.3)	1.3 (1.3)

WHO-5 (score)	63.5 (22.9)	60.9 (24.1)	61.4 (23.7)	61.1 (24.0)

BMI, body mass index; TMIG-IC, Tokyo Metropolitan Institute of Gerontology Index of Competence; GDS, Geriatric Depression Scale; WHO-5, World Health Organization Five Well-Being Index.

### 3.2 Main analyses

During the follow-up period of approximately 4 years, 5.0 % had incident disabling dementia. Among current dog owners and past and never dog owners, 3.6 % and 5.1 % had incident disabling dementia, respectively. Among current cat owners and past and never cat owners, the corresponding values were 4.5 % and 5.0 %. Results showed that current dog owners had an OR of 0.60 (95 %CI: 0.37–0.97) of disabling dementia compared to past and never owners. For cat ownership, the corresponding OR was 0.98 (95 %CI: 0.62–1.55) (Table2).

**Table 2:** Independent associations between experience with dog/cat ownership and incident dementia among community-dwelling older Japanese.

Independent variable	Incident dementia (n = 11194)	Propensity score in GEE, OR (95 %CI) (n = 8323 for dog ownership and n = 9579 for cat ownership)
Experience with dog ownership		
Past and never (n = 10235, 91.4 %) §	525/10235 (5.1 %)	1
Current (n = 959, 8.6 %)	35/959 (3.6 %)	0.60 (0.37–0.97)

Experience with cat ownership		
Past and never (n = 10490, 93.7 %) §	528/10490 (5.0 %)	1
Current (n = 704, 6.3 %)	32/704 (4.5 %)	0.98 (0.62–1.55)

§ reference group

GEE, generalized estimating equation; OR, odds ratio; CI, confidence interval.

Analysis was weighted by the inverse of propensity score in the GEE. Weight was calculated based on sex, age, household size, marital status, educational attainment, equivalent income, employment, history of chronic diseases (lung respiratory disease, and cancer), history of hospitalization during the past year, fall during the past year, alcohol consumption, smoking status, TMIG-IC score, mobility limitation, motor fitness scale, frailty status, interaction with neighbors, social isolation, trust in neighbors, subjective happiness, and WHO-5 for dog ownership. For cat ownership, weight was calculated based on sex, age, household size, marital status, educational attainment, equivalent income, employment, fall during the past year, smoking status, TMIG-IC score, interaction with neighbors, and social isolation. Adjusted for follow-up period.

### 3.3 Sensitivity analysis

Inverse probability of treatment weighted logistic regression model with the propensity score excluded events in the first year showed that dog owners had an OR of 0.67 (95 %CI: 0.40–1.14) of disabling dementia compared to past and never owners.

### 3.4 Additional analyses

**Fig. 1:**
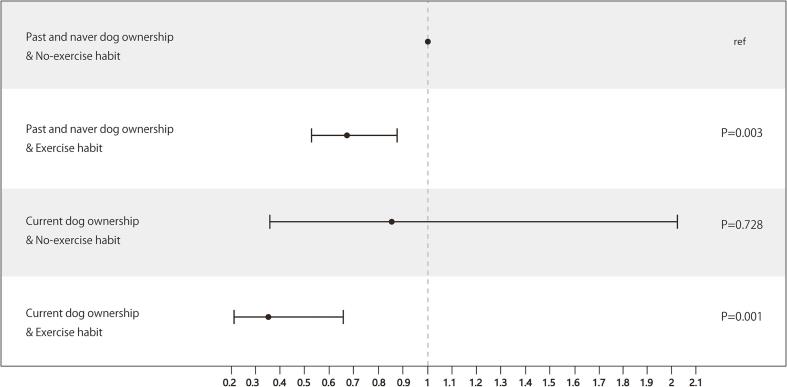
Odds ratios of dog ownership and habitual exercise with incident dementia. An inverse probability of treatment weighted logistic regression model with the propensity score was implemented. X-axis showed odds ratios.

**Fig. 2:**
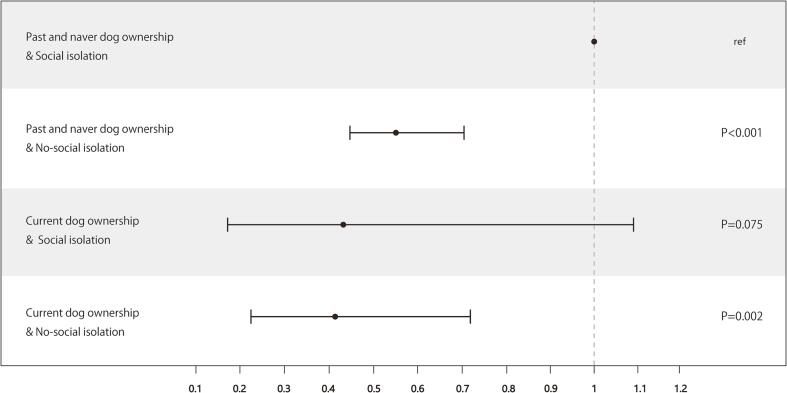
Odds ratios of dog ownership and social isolation with incident dementia. An inverse probability of treatment weighted logistic regression model with the propensity score was implemented. X-axis showed odds ratios.

Examining the association of the interaction between dog ownership and exercise habit with incident disabling dementia showed that current dog owners with a regular exercise habit had an OR of 0.37 (95 %CI: 0.20–0.68), current dog owners with no exercise habit had an OR of 0.89 (95 %CI: 0.36–2.03), and past and never dog owners with an exercise habit had an OR of 0.69 (95 %CI: 0.54–0.88) compared to past and never dog owners with no exercise habit (Fig. 1). Further, analysis of the association of the interaction between dog ownership and social isolation with incident disabling dementia revealed that current dog owners with no social isolation had an OR of 0.41 (95 %CI: 0.23–0.73), current dog owners with social isolation had an OR of 0.43 (95 %CI: 0.17–1.09), and past and never dog owners with no social isolation had an OR of 0.56 (95 %CI: 0.45–0.71) compared to past and never dog owners with social isolation (Fig. 2).

## 4 Discussion

Our previous studies demonstrated that dog ownership has a protective effect on frailty (Taniguchi et al., 2019), disability and death (Taniguchi et al., 2022). The present study is the first to show that dog ownership has a protective effect on incident disabling dementia. Dog owners had an OR of 0.60 of incident disabling dementia after adjusting for background factors during an approximately 4-year follow-up period compared to non-dog owners. This association was confirmed using sensitive analysis in which an inverse probability of treatment weighted logistic regression model with the propensity score excluded events in the first year. The present study also revealed that dog owners with an exercise habit and no social isolation have a significantly lower risk of disabling dementia.

Here, we discuss the mechanism that underlies the association of dog ownership with incident disabling dementia. This study showed that physical activity, including having an exercise habit and social participation through daily dog care can prevent dementia in older adults. Dog walking is categorized as a physical activity with 3 metabolic equivalents of moderate intensity (National Institute of Health and Nutrition., 2012) and dog owners who walk their dog had 2.5 times more likely to achieve at least moderate-intensity physical activity of 150 mins/week (Soares et al., 2015). Dog walking has also been implicated as a means to increase opportunities for social interaction and improve psychological health for older adults (Carr et al., 2021, Ikeuchi et al., 2021), suggesting that dog walking may contribute to social participation. Lower physical activity (Sofi et al., 2011) and lower social participation (Kuiper et al., 2015) are associated with higher rates of cognitive decline and dementia (World Health, 2019). In the present study, regardless of experience with dog ownership, exercise habit and no social isolation had lower OR of incident disabling dementia, which consistent with the above previous studies. We propose that these factors (physical activity, including having an exercise habit, and social participation) underlie the beneficial relationship between dog ownership and incident dementia. Meanwhile, dog owners without daily lifestyle habits related to dog care, such as no exercise habit and social isolation, did not experience positive effects related to dementia prevention. Likewise, cat ownership was not effective for preventing dementia. This result largely overlaps with previous studies on frailty (Taniguchi et al., 2019), disability and death (Taniguchi et al., 2022).

Several types of strategies, such as exercise and social interaction programs, have been implemented around the world to prevent dementia and the need for long-term care. The present study suggests that daily physical and social activity related to dog care may be effective for dementia prevention. A previous study reported that dog owners had higher well-being during the COVID-19 pandemic than owners of other pets (Amiot et al., 2022). Therefore, dog care might contribute to the maintenance of physical activity, including having an exercise habit and social participation in the face of restrictions to interactions such as those experienced during the COVID-19 pandemic. Moreover, a social support system may be necessary for older adults to continue to own and care for a dog or other pet.

This study has several main strengths. First, our large sample of community-dwelling older Japanese and the availability of sufficient prior evidence (Taniguchi et al., 2018b) enabled us to perform propensity score matching. The present study adjusted for 22 and 12 variables related to the backgrounds of dog and cat owners in Table 1, respectively, to examine the association of dog/cat ownership with incident disabling dementia. Second, data on incident disabling dementia were derived from the LTCI system database. The universal LTCI system in Japan enabled us to link dog ownership with incident disabling dementia.

However, this study also has some limitations. First, the proportion of dog and cat owners in Japan is smaller than that in Western countries. It will thus be important to assess whether the relationships found in Japan are also present in Western and other countries. Second, based on available evidence (Taniguchi et al., 2022), this study focused on pathways related to exercise habit and social isolation in the relationship between dog ownership and disabling dementia. Future studies should consider the psychological pathways that may link dog ownership to reduced dementia onset. Third, we used the LTCI system to assess incident disabling dementia until July 2020. Because of the first wave of COVID-19 infections in Japan, the number of new applications for LTCI drastically decreased from March to May 2020 (Seino et al., 2021). Therefore, the number of participants with incident disabling dementia during follow-up may have been underestimated, along with the association between dog ownership and disabling dementia. Finally, we did not examine cognitive function (Taniguchi et al., 2017a) in the baseline survey, and we assessed incident disabling dementia over a relatively short follow-up period of 4 years. These two limitations have the possibility to lead to reverse causality. Thus, further study is needed to address these limitations and confirm the association between dog ownership and incident disabling dementia.

## 5 Conclusion

This prospective study revealed that dog ownership had a suppressive effect on incident disabling dementia after adjusting for background factors during an approximately 4-year follow-up period. Specifically, dog owners with an exercise habit and no social isolation had a significantly lower risk of disabling dementia. Dog care might contribute to the maintenance of physical activity, including having an exercise habit, and social participation even in the face of restrictions to interactions such as those experienced during the COVID-19 pandemic.

## Funding

This study was supported by grants from Ota City, Japan Health Promotion & Fitness Foundation, Sugiura Memorial Foundation, The Japan Foundation for Aging and Health, and JSPS KAKEN Grant number 16K16615. The funders played no role in the study design, data collection and analysis, decision to publish, or preparation of the manuscript.

## CRediT authorship contribution statement

**Yu Taniguchi:** Conceptualization, Formal analysis, Writing – original draft, Writing – review & editing. **Satoshi Seino:** Conceptualization, Funding acquisition, Investigation, Resources, Writing – review & editing. **Tomoko Ikeuchi:** Conceptualization, Writing – original draft, Writing – review & editing. **Toshiki Hata:** Investigation, Resources, Writing – review & editing. **Shoji Shinkai:** Funding acquisition, Investigation, Supervision, Writing – review & editing. **Akihiko Kitamura:** Funding acquisition, Investigation, Supervision, Writing – review & editing. **Yoshinori Fujiwara:** Funding acquisition, Investigation, Supervision, Writing – review & editing.

## Declaration of Competing Interest

The authors declare that they have no known competing financial interests or personal relationships that could have appeared to influence the work reported in this paper.

## Data Availability

Data will be made available on request.
